# Therapies with Antioxidant Potential in Psoriasis, Vitiligo, and Lichen Planus

**DOI:** 10.3390/antiox10071087

**Published:** 2021-07-06

**Authors:** Fabrizio Guarneri, Lucrezia Bertino, Giovanni Pioggia, Marco Casciaro, Sebastiano Gangemi

**Affiliations:** 1 Department of Clinical and Experimental Medicine, Dermatology, University of Messina, 98125 Messina, Italy; fabrizio.guarneri@unime.it (F.G.); bertino.lucrezia@gmail.com (L.B.); 2 Institute for Biomedical Research and Innovation (IRIB), National Research Council of Italy (CNR), 98164 Messina, Italy; giovanni.pioggia@cnr.it; 3 School and Unit of Allergy and Clinical Immunology, Department of Clinical and Experimental Medicine, University of Messina, 98125 Messina, Italy; gangemis@unime.it

**Keywords:** antioxidants, oxidative stress, inflammation, skin diseases, vitiligo, psoriasis, lichen planus

## Abstract

Oxidative stress plays an important pathogenetic role in many chronic inflammatory diseases, including those of dermatological interest. In particular, regarding psoriasis, vitiligo, and lichen planus, excess reactive oxygen species and a decline in endogenous antioxidant systems are observed. In this regard, treatments with antioxidant properties could be appropriate therapeutic options. To date, clinical trials in dermatology on these treatments are limited. We reviewed the available studies on the efficacy of antioxidant therapies in psoriasis, vitiligo, and lichen planus. The role of herbal derivatives, vitamins, and trace elements was analyzed. The antioxidant properties of conventional therapies were also evaluated. Data from the literature suggest that antioxidants might be useful, but available studies on this topic are limited, heterogeneous, not completely standardized, and on small populations. Furthermore, in most cases, antioxidants alone are unable to induce significant clinical changes, except perhaps in mild forms, and must be used in conjunction with standard drug treatments to achieve measurable results. Further studies need to be conducted, considering larger populations and using internationally validated scales, in order to compare the results and clinical efficacy.

## 1. Introduction

### 1.1. Inflammatory Skin Diseases

The pathogenesis of inflammatory skin diseases is complex, and many mechanisms have yet to be clarified. Emerging evidence suggests that genetic and epigenetic factors influence the inflammatory response [1]. Environmental factors also seem to contribute to activation of the innate and adaptive immune system via the production of pro-inflammatory cytokines [2]. When inflammation becomes chronic, overproduction of reactive oxygen species (ROS) such as superoxide (O_2_^−^) and hydrogen peroxide (H_2_O_2_) can be observed [3].

### 1.2. Oxidative Stress Involvement

The major sources of endogenous ROS are mitochondria, NADPH oxidase, cyclooxygenases, lipoxygenases, and cytochrome P450. ROS production can also be stimulated by exogenous factors (e.g., sun exposure). Sometimes ROS production increases and exceeds the capacity of the antioxidant system. This phenomenon is defined as oxidative stress (OS), whose impact on biological systems can be detected by some biomarkers. In particular, malondialdehyde (MDA) and 4-hydroxy-2-nonenal are end products of lipid peroxidation, and oxidative and carbonyl stress lead to the permanent production of oxidation products, such as advanced glycation end products (AGEs) and advanced oxidation protein products (AOPPs), due to the conversion of excess metabolites such as glucose and lipids.

Emerging evidence suggests that OS is involved in the pathophysiological pathways of several disorders, such as cardiovascular diseases, retinal degeneration, benign prostatic hyperplasia, diabetic disease, asthma, and neurodegenerative diseases [4,5,6]. As mentioned above, OS also plays a role in the pathogenesis of chronic skin diseases [7,8] and in the transformation and progression of skin cancers, in particular melanoma [9].

### 1.3. Psoriasis

Psoriasis (PSO), vitiligo, and lichen planus (LP) are three common inflammatory skin diseases with complex pathogenesis. PSO is a chronic relapsing, immune-mediated disease characterized by red and scaly patches on the skin. Histologically, hyperproliferation and aberrant differentiation of keratinocytes and infiltration of inflammatory cells can be observed. In PSO patients, as demonstrated by many studies, detrimental external triggers such as cigarette smoking, air pollution, physical damage, and biological agents (viruses, bacteria, etc.) can contribute to keratinocyte damage [10,11]. Consequently, breaches in skin cell membranes lead to the release of inflammatory mediators and alarmins [12]. Alarmins are molecules that are normally present in some contexts, such as cytoplasm, but act as pro-inflammatory attractants when present in blood or tissue. For this reason, alarmins are also known as danger signals [13]. Together, inflammatory mediators and alarmins sustain inflammation, which consequently leads to ROS generation, creating a vicious cycle that is fundamental in PSO pathogenesis [14]. ROS can cause damage to many biomolecules in specific processes, including lipid peroxidation, and can stimulate the secretion of proinflammatory cytokines [15]. Other studies have demonstrated that a lack of enzymes or alterations of enzymes involved in the regulation of the oxidoreductive balance, such as glutathione S-transferase (GST) M1/T1 polymorphisms, may play a role in the complex pathogenetic mechanism of psoriasis, by reducing the antioxidant potential of the organism [16].

### 1.4. Vitiligo

Vitiligo is a chronic autoimmune and autoinflammatory cutaneous disease that causes depigmentation [17]. Vitiligo results from interactions between genetic, biochemical, environmental, and immunological factors, and OS is an important pathogenetic element. The epidermis is constantly exposed to environmental stressors, which can increase ROS production. Melanocytes of vitiligo patients are incapable of handling these stressors, resulting in dilated endoplasmic reticulum and abnormalities in the mitochondria and melanosome structure. Moreover, vitiligo patients have elevated concentrations of AOPPs and AGEs [18] and epidermal H_2_O_2_, and decreased levels of catalase (CAT) [19]. In addition, recent reports suggest that inflammation induces the release of interleukin (IL)-33 and IL-23, which, in turn, triggers a Th2 response and drives a proinflammatory loop [20,21].

### 1.5. Lichen Planus

Lichen planus (LP) is a chronic inflammatory and immune-mediated disease that may affect the skin, cutaneous adnexa, and/or mucous membranes. Many variants of LP exist, differentiated on the basis of the morphology of lesions and body site(s) involved. The disease can negatively affect the quality of life of patients, particularly forms such as hypertrophic LP and erosive oral LP. Several risk factors have been described in the etiology of LP, including hepatitis C virus infection, drug use, and emotional stress [22,23]. The inflammatory infiltrate consisting of T lymphocytes contributes to local cytokine production and cell damage. As a consequence of cytokine stimulation, ROS are generated, triggering keratinocyte apoptosis [24,25].

### 1.6. Oxidative Stress and Immune System

OS, defined as a pro-oxidant vs. antioxidant imbalance in favor of the first [26], plays an important role in all of the above inflammatory skin diseases [27]. Skin is the barrier organ that protects the human body from external threats. For this reason, it is subjected to constant exposure to ROS, which are responsible for keratinocyte damage. Cell damage leads to the release of pro-inflammatory mediators and recruitment of immune cells. When the endogenous antioxidant system fails to adequately reduce OS [28], the oxidized molecules that are released after tissue damage and augmented due to the inflammation stimulate a detrimental loop. The disruption of redox signaling and control takes on primary importance in the pathogenesis of many diseases [29]. The key role of immune cells in OS balance has been confirmed, as reported by Wang et al., who highlighted the phagocytosis, degranulation, and ROS generation acted on by neutrophils in psoriasis [30]. Based on recent discoveries, oxidation and immune system activation and regulation appear to be intimately linked [31].

### 1.7. Antioxidants

In this context, a form of treatment with antioxidant properties could be a therapeutic option. Antioxidants are substances that neutralize ROS, preventing cell and tissue damage. Based on their activity, antioxidants are classified as either non-enzymatic or enzymatic [32,33]. The first group includes exogenous non-enzymatic molecules such as vitamins E, A, and C, flavonoids, carotenoids, plant polyphenols, theaflavin, allyl sulfides, selenium, and curcumin [34]. Endogenous non-enzymatic molecules include melatonin, bilirubin, uric acid, polyamines, and glutathione (GSH). GSH belongs to the glutathione system, which includes the enzymes glutathione reductase, glutathione peroxidase (GPX), and glutathione-S-transferase (GST) [34]. Enzymatic antioxidants also include superoxide dismutase (SOD), superoxide reductase, CAT, and thioredoxin [35]. When there is excess ROS and a decline in the endogenous antioxidant system, such as in inflammatory skin diseases, ingestion or topical application of antioxidant molecules could theoretically be indicated [32,33]. To date, clinical trials in dermatology on these treatments are limited. The aim of this review is to summarize the available studies on the efficacy of antioxidant therapies in PSO, vitiligo, and LP, and to discuss potential future treatment options.

## 2. Material and Methods

This review was performed by browsing the PubMed database (http://www.pubmed.gov, last accessed date on May 2021), searching from inception until 31 March 2021. The search strategy included terms related to inflammatory cutaneous diseases (“psoriasis”, “vitiligo”, and “lichen planus”) and antioxidant treatment (“antioxidant therapy” and “antioxidant treatment”).

Articles were included if they: (a) reported on clinical trials that (b) were conducted on humans and not other species; (c) actually included patients affected by vitiligo or PSO or LP; (d) clearly reported the demographic and clinical characteristics of the studied population; (e) included patients treated with therapeutic options that act on redox status; and (f) were written in English.

We read the abstracts of articles whose titles suggested that the association between the aforementioned inflammatory skin diseases and antioxidant therapy was analyzed. We read the entire article only if the abstract indicated that it potentially met the inclusion criteria. Finally, we reviewed and searched the references of these articles in order to identify further studies that could be included. Data obtained from the studies included author names, publication date, study design, number of patients, severity of skin disease or type of lesion, chosen treatment, treatment formulation and adverse effects, duration of treatment exposure, and degree of disease improvement.

## 3. Results

The initial PubMed search yielded 585 articles involving patients with PSO, 257 articles involving patients with vitiligo, and 68 articles involving patients lichen planus. Of these, 544 on PSO, 232 on vitiligo, and 56 on LP were not considered as: (a) the title and/or abstract suggested that they were not clinical trials, (b) they were not written in English, and (c) the trials were not performed on only human populations. We then read the full text of the remaining 41, 25, and 12 articles, respectively, and selected 17, 18, and 7 of these, considering the content and the relevance to the outcome of interest.

### 3.1. Psoriasis

We selected 17 studies on therapy with antioxidant effects in PSO: seven described oral antioxidant supplementation, two described topical antioxidant treatment, and two pointed out the effects of physical treatment, four of systemic therapy, and two of biologic therapy on redox status (Table 1).

#### 3.1.1. Topical Antioxidant Therapy

The clinical effects of topical curcumin were reported in two clinical trials. Curcumin is a strong antioxidant, and is the strongest active ingredient in the root of the Curcuma plant (*Curcuma longa*). One group of authors tested the effects of topical curcumin on psoriasis. They observed an improvement in lesions and an inhibition of phosphorylase kinase activity [36]. Bahraini et al. investigated the efficacy of turmeric tonic in patients with mild to moderate scalp PSO. Half of the patients received tonic twice a day for 9 weeks, while the other half received a placebo. Compared to placebo, PASI score was significantly reduced in the first group, improving quality of life (*p* < 0.05) [37].

#### 3.1.2. Oral Antioxidant Supplementation

Five studies described the effects of oral curcumin in PSO patients; in the majority of cases, significant improvement of the disease was observed.

Antiga et al. assessed the superiority of topical methylprednisolone aceponate (a synthetic glucocorticoid) plus oral curcumin (2 g/day for 12 weeks) over topical methylprednisolone plus placebo in patients with moderate to severe PSO [38]. They evaluated the efficacy of the treatments with PASI, and they collected blood samples to determine IL-17 and IL-22 serum levels. Although both groups achieved a significant reduction in PASI values, the reduction was greater in patients treated with oral curcumin (*p* < 0.05). In addition, after 12 weeks of treatment, IL-22 levels showed a significant reduction in this arm of the study (*p* < 0.001), while no differences were observed in IL-17 levels.

In two studies, oral curcumin (600 mg/day) plus either UVA radiation or visible blue light was administered [39,40]. In both studies, 100% of the patients were responders, but in one study the local response was considered better in patients treated with visible light compared to those treated with simulated visible light. Another group of researchers compared the efficacy of visible blue light plus oral curcumin (600 mg/day for 12 weeks) versus psoralen plus UVA therapy (PUVA). They observed a slower response in the first arm. The data also suggested that oral curcumin plus visible light had the same efficacy as PUVA, but a safer profile [41]. On the other hand, Kurd et al. showed that oral curcumin (4.5 g/day) yielded a low response rate in 12 patients with moderate to severe psoriasis [42].

Some authors investigated the efficacy of combinations of different antioxidant molecules on redox status. Kharaeva et al. evaluated the clinical effects of supplementation with coenzyme Q10 (ubiquinone acetate 50 mg/day), vitamin E (natural α tocopherol 50 mg/d), and selenium (Se; aspartate salt 40 µg/d) in two groups: 14 patients with erythrodermic PSO (EP) and 15 patients with psoriatic arthritis (PsA). The authors assessed markers of OS at baseline and, after supplementation, every 5 days for a total of six times; the activities assessed were OS production, copper/zinc ratio (Cu/Zn), SOD, and CAT activity in the circulating granulocytes and the epidermis, and plasma levels of nitrites/nitrates. They found that supplementation resulted in faster improvement of clinical symptoms compared to the placebo group (24 healthy donors matched by age and sex). In particular, clinical improvement corresponded to a normalization of all OS markers analyzed [43].

In another clinical trial, Fairris et al. studied the effect of supplementation with Se and vitamin E in PSO patients [44]. They recruited 69 patients, allocating them to three groups: 22 patients received 600 µg of Se daily, 23 patients received 600 µg of Se plus 600 IU of vitamin E daily, and 24 patients received placebo tablets. Patients were age- and sex-matched with healthy controls. The supplementation was taken for 12 weeks. Every 4 weeks, the PASI score was calculated and blood samples were taken to assess plasma Se and vitamin E status, red cell Se and GPX status, and white cell Se status. Skin biopsies were also taken at the beginning of the study and the end of week 12 to evaluate skin Se concentration and GPX activity. At baseline, Se concentration was reduced compared to healthy subjects (matched for age, sex, use of contraceptive pills, and number of cigarettes smoked), but after supplementation it raised significantly, together with GPX activity and vitamin E concentration. Despite these phenomena, skin Se and GPX activity remained unchanged. In fact, the authors did not observe a reduction in the severity of PSO.

#### 3.1.3. Physical Therapy

Two different groups of authors studied the role of narrowband ultraviolet B (NB-UVB) on the redox status in PSO patients. Wacewicz et al. determined the concentrations of Se, Zn, and Cu and the Cu/Zn ratio, as well as total antioxidant status and C-reactive protein in the serum of 60 PSO patients before and after 20 sessions of NB-UVB. The control group consisted of 58 healthy volunteers without any sign of skin disease, matched to study subjects by sex and age. PASI score and serum Se concentration after NB-UVB were lower in PSO patients compared to controls (*p* < 0.05). On the other hand, Cu levels were higher compared to controls (*p* < 0.05). No significant differences were found in total antioxidant status and Zn levels, but a higher Cu/Zn ratio (*p* < 0.05) in PSO patients than in the control group was observed [45].

Darlenski et al. addressed the link between disease activity, epidermal barrier, and systemic oxidative stress in PSO patients treated with 14 sessions of NB-UVB. At baseline and at treatment completion, blood samples were collected to assess MDA, ROS, ascorbyl radicals, and CAT detoxifying activity. The authors also measured local trans-epidermal water loss and stratum corneum hydration. A significant improvement in disease activity was observed (*p* < 0.0001). Moreover, the significant clinical response was reflected in better epidermal barrier function (less trans-epidermal water loss and higher stratum corneum hydration compared to baseline) and improved systemic oxidative stress parameters (ROS, MDA, and ascorbyl radicals) (*p* < 0.0001) [46].

#### 3.1.4. Systemic Therapy

Akbulak et al. evaluated the beneficial role of methotrexate (MTX) in the expression of isoenzymes of GST and cytochrome families. They included 21 PSO patients treated with MTX 10–15 mg/week for a minimum of 12 weeks and 22 healthy subjects. They found significantly higher tissue levels of GST and cytochrome expression in PSO patients than in controls, but they also found that MTX did not have a significant effect on these parameters [47].

Köse et al. evaluated the effect of oral propylthiouracil (PTU), a treatment with antioxidant potential and immunomodulatory effect, in psoriatic patients. They determined SOD and GPX activity and MDA levels in plasma, erythrocytes, and skin biopsies in two groups; 20 psoriatic patients were treated with oral PTU 100 mg three times daily, and 10 patients were treated with PTU plus 25 µg of thyroxine daily (to prevent possible hypothyroidism, which can be induced by PTU) for 8 weeks. Treatment resulted in significantly lower PASI scores in both groups (*p* < 0.01). Moreover, erythrocyte, plasma, and tissue MDA levels were significantly decreased in the first group, while erythrocyte and tissue SOD levels and GPX activity were higher in PSO patients in both groups, without significant difference between them [48].

Clinical improvement with PTU was observed by Elias et al. in two trials. In the first trial, the authors pointed out the usefulness of treating PSO patients with oral PTU 300 mg/day for 8 weeks [49]. PASI score fell from an initial value of 21.04 ± 4.10 to 12.95 ± 3.16 (*p* < 0.004). A significant improvement in PSO was also described with topical application of 60 g of PTU in 5% lotion twice a week for 4–8 weeks (*p* < 0.02 at 8 weeks vs. age- and sex-matched controls). Placebo-treated and untreated areas showed no significant change during the study [50].

#### 3.1.5. Biologic Therapy

Two clinical trials investigated whether the modulation of inflammatory activity by tumor necrosis factor α inhibitors (TNFα-I) was associated with modification of oxidative stress status. Bacchetti et al. evaluated plasma lipid and lipoprotein a levels and inflammatory and lipid peroxidation markers in PSO patients before and after 24 weeks of treatment with etanercept [51]. They also analyzed plasma total antioxidant capacity (TAC) and the activity of paraoxonase (PON) 1, a hydrolytic enzyme that can protect against lipid oxidation. Significant clinical improvement was observed in association with reduced levels of inflammatory markers and lipid peroxidation, and increased serum TAC (*p* < 0.001). Moreover, the study showed a significant increase in PON1 activity (*p* < 0.001).

Barygina et al. investigated the effects of the TNFα-I infliximab on blood redox status in PSO patients. At the beginning and the end of the study, the authors evaluated the levels of main oxidative stress markers, TAC, lipoperoxidation, GSH content, and NADPH oxidase activity in plasma or white blood cells. Plasma MDA and protein carbonyl content levels were significantly lower in the PSO patients than in the untreated group, while NADPH oxidase activity was significantly increased in white blood cells (*p* < 0.05) [52].

### 3.2. Vitiligo

For vitiligo, we selected 18 studies: 6 studies described the efficacy of oral antioxidant supplementation, 11 described topical antioxidants, and 2 analyzed the effects of topical calcineurin inhibitors on redox status (Table 2).

#### 3.2.1. Oral Antioxidant Supplementation

Two groups of authors determined the efficacy of oral antioxidant supplementation with NB-UVB. Elgoweini et al. recruited 24 patients and divided them into two groups: one group was treated with alpha-tocopherol (vitamin E) 400 IU once daily plus NB-UVB, and the second with phototherapy alone [53]. They also measured plasma MDA and GSH before and after treatment. The authors observed a significant reduction in plasma MDA (*p* < 0.001) and excellent repigmentation in 72.7% of patients in the first group, compared to 55.6% in the second group. Moreover, erythema related to phototherapy was less frequent in the first group. In another trial, 28 patients received daily treatment of two tablets containing α-lipoic acid (50 mg), vitamin C (50 mg), vitamin E (20 mg), polyunsaturated fatty acids (12%), and cysteine monohydrate (50 mg), in addition to phototherapy. This group of patients was compared with a placebo group including 14 patients [54]. After 6 months of therapy, antioxidant supplementation increased the therapeutic success of phototherapy, with 47% of patients obtaining > 75% repigmentation vs. 18% in the placebo group (*p* < 0.05). Moreover, it significantly reduced vitiligo-associated oxidative stress: catalase activity increased to 114% (*p* < 0.05 vs. placebo) and ROS level decreased up to 60% (*p* < 0.02 vs. placebo).

Four studies analyzed the efficacy of herbal derivatives as antioxidant therapy. Colucci et al. investigated the role of an oral supplement containing *Phyllanthus emblica* (100 mg), vitamin E (4.7 mg), and carotenoids (10 mg) [55]. In that study, 65 patients took one tablet three times a day for 6 months, while a second group of 65 patients received no supplementation; they all continued any concomitant vitiligo treatments. The first group had only mild repigmentation (*p* < 0.05); however, the number of patients without improvement was higher in the second group, suggesting that oral supplementation might be a valid tool to increase the effectiveness of other vitiligo treatments. In another study, the efficacy of oral supplementation with extracts from *Ginkgo biloba* was evaluated [56]. In that study, 52 patients were divided into two groups: 26 took *G. biloba* extract 40 mg thrice daily and 26 took a placebo, for a 6-month treatment period. In the first group, a significant cessation of depigmentation was noted (*p* = 0.006), and marked to complete repigmentation was observed in about 40% of cases. Taken together, these data suggest that *Ginkgo biloba* extract seems to be a fairly effective therapy for arresting the progression of vitiligo. Middelkamp-Hup et al. studied whether supplementation with *Polypodium leucotomos* would improve repigmentation induced by NB-UVB, the first choice of treatment for vitiligo. They enrolled 50 patients, who randomly received 250 mg of *p. leucotomos* or placebo orally three times daily, combined with NB-UVB twice weekly for 25–26 weeks. The authors observed a greater amount of repigmentation in the treatment group compared to the placebo group, with nearly statistical significance (*p* = 0.06). Areas with a better response were the neck and head [57]. Finally, a group of authors examined the effect of phototherapy plus oral *Silybum marianum* in vitiligo patients. They observed significant improvement in repigmentation in patients treated with both antioxidant therapy plus NB-UVB and NB-UVB alone. However, a better response was seen in the first group [58].

#### 3.2.2. Topical Antioxidant Therapy

Schallreuter et al. analyzed the effect of NB-UVB-activated pseudocatalase cream (PC-KUS) in combination with climatotherapy at the Dead Sea in patients with vitiligo [59]. Pseudocatalase is a complex of bis-Mn*^III^* [EDTA]_2_ [HCO_3_^−^]_2_ which mimics the effect of catalase. Convincing evidence indicates that, in vitiligo skin, there are high levels of epidermal H_2_O_2_ and low catalase levels, which could be increased by PC-KUS. In a previous study, the authors obtained excellent repigmentation in patients with focal vitiligo or facial and dorsum vitiligo with PC-KUS plus NB-UVB [60]. In the more recent trial, they observed significantly faster initiation of repigmentation compared to conventional PC-KUS monotherapy or climatotherapy alone (*p* = 0.0001). On the contrary, Patel et al. did not observe clear evidence for the efficacy of pseudocatalase mousse applied twice daily plus NB-UVB phototherapy twice weekly for a period of 24 weeks [61]. In another trial, patients applied this cream for 24 weeks in combination with NB-UVB (three times a week). These authors also pointed out that pseudocatalase cream plus NB-UVB did not show statistically significant improvement compared to NB-UVB alone [62].

Kostovic et al. studied the efficacy of a topical gel containing CAT as well as SOD [63]. In that study, 22 patients applied the gel twice daily for a 6-month period, and also received narrowband UVB three times per week. In 57.9% of patients, more than 50% repigmentation was obtained, with the best response on the face and neck, while lesions on the hands and feet were the most refractory. In a previous study, Schallreuter et al. reported that this gel did not show any significant improvement in six patients with facial vitiligo who applied the formulation twice daily and underwent solar exposure for at least 30 min over 4 months [64]. Yuksel et al. reported no statistically significant difference (*p* > 0.05) between 15 patients treated with NB-UVB alone and 15 patients treated with phototherapy and a topical formulation including *Cucumis melo* SOD and CAT for 6 months [65]. Another study evaluated the efficacy of a similar gel containing phenylalanine, *Cucumis melo* extract, and acetyl cysteine, given alone or in combination with 311 nm narrowband microphototherapy. They compared that group of patients with two other groups of patients, one treated with microphototherapy alone and one with clobetasol propionate (a synthetic glucocorticoid) 0.05% ointment alone. Excellent repigmentation (>75%) was achieved in about 40% of patients treated with gel alone. Significant improvement of lesions was obtained in patients treated with gel and phototherapy [66]. Sanclemente et al. compared the efficacy of topical CAT plus SOD with topical 0.05% betamethasone combined with solar exposure for at least 15 min [67]. Interestingly, they pointed out similar repigmentation with these two therapies, with the further advantage of milder adverse effects with topical catalase plus superoxide dismutase compared to topical corticosteroids.

Other authors evaluated the efficacy of topical SOD combined with excimer light therapy. The combination therapy was more efficient than excimer light therapy alone (*p* < 0.001) [68]. Leone et al. evaluated the combined effect of excimer laser and a topical antioxidant cream containing folic acid, phenylalanine, sitosterol, hyaluronic acid, and a proprietary combination of substances (extract of Mexican bamboo, boldine from the Chilean boldo tree, aminoguanidine HCl, and decarboxy carnosine HCl) as active ingredients [69]. Ten patients applied the product twice daily for 3 months in combination with twice weekly laser treatment. Notably, the results indicated that repigmentation degree and speed were, respectively higher and slower than with laser treatment alone. These findings suggest that this cream could shorten the course of phototherapy, ultimately minimizing the possible side effects of UV irradiation of the skin.

#### 3.2.3. Topical Calcineurin Inhibitors

Lubaki et al. analyzed the effect of topical immunomodulators, such as tacrolimus and pimecrolimus, on repigmentation of vitiligo lesions. They also evaluated the redox status before and after 7 months of topical treatment, assessing derivatives of oxygen metabolites in the serum of 22 patients. More significant repigmentation was observed on the face compared to other locations. Moreover, tacrolimus showed an ability to reduce oxidative status and increase antioxidant capacity independently from its indirect capacity for repigmentation [70].

### 3.3. Lichen Planus

With regard to lichen planus, seven studies were selected: three analyzed the effects of oral antioxidant supplementation in oral lichen planus (OLP), and four analyzed the effects of topical antioxidant therapy (Table 3).

#### 3.3.1. Oral Antioxidant Supplementation

One clinical trial was conducted to assess the efficacy of herbal derivatives, which may contain numerous biologically active compounds. Agha-Hosseini et al. evaluated the effectiveness of purslane, an herbaceous weed that contains several compounds, including vitamins A, C, and E, melatonin, β-carotene, omega-3 fatty acids, and minerals [71]. It seems to possess anti-inflammatory, anti-ulcerogenic, antifungal, and antioxidant properties. In that study, 37 patients with OLP were divided into two groups to receive 235 mg of purslane or placebo, orally, for 3 months. At baseline, after 2 weeks, and each month for 6 months, the authors assessed a visual analogue scale (VAS) and clinical improvement of lesions. Approximately 83% of the treated patients experienced partial to complete clinical response, but 17% had no improvement, showing a significant difference compared with the placebo group (*p* < 0.001). Partial to complete symptomatic responses were observed in all purslane-treated patients (*p* < 0.001).

Saawarn et al. assessed the efficacy of systemic administration of lycopene, a carotenoid with antioxidant activity that can inhibit cancer cell proliferation, in OLP patients [72]. They recruited 30 patients, 15 of whom were treated with lycopene 8 mg/day, and 15 with placebo, for 8 weeks. Compared to the control group, patients treated with lycopene had a higher (84%) reduction in burning sensation. Moreover, all 15 patients in the lycopene group showed 50% or more clinical response, and 11 of them (73.3%) showed 70–100% response.

Finally, Qataya et al. described the efficacy of Se in two forms (oral capsules and topical hydrogel) for treating erosive OLP, comparing these two treatments with topical corticosteroids [73]. Patients were evaluated at baseline, and after 6 and 12 weeks. Salivary MDA and TAC levels were evaluated at baseline and 6 weeks. Signs and symptoms were significantly reduced in all treatment modalities. However, there was no significant difference among the three groups at 6 weeks. At 12 weeks, patients treated with topical Se had significantly lower pain scores compared with patients treated with topical steroids. Salivary MDA levels showed a significant decrease in patients with topical treatment; no significant difference was observed in TAC levels.

#### 3.3.2. Topical Antioxidant Therapy

Bacci et al. assessed the efficacy of topical tocopherol acetate compared with placebo in 34 patients with reticular OLP [74]. Patients were divided into two groups (A or B), and each group received two treatments sequentially (group A: tocopherol then placebo; group B: placebo then tocopherol) for 2 months, with a 2-week washout period between them. In group A, compared with group B, a significant reduction in surface area of lesions (*p* = 0.0045) and lower modified Thongprasom scores (*p* = 0.0052) were observed, while no differences were seen in length of striae and VAS scores. The Thongprasom score is a clinical score where 0 = no lesions; 1 = white striae with no signs of erythema; 2 = white striae with areas of atrophy <1 cm^2^; 3 = white striae with areas of atrophy >1 cm^2^; 4 = white striae with areas of erosion <1 cm^2^; and 5 = white striae with areas of erosion >1 cm^2^.

Two other articles described the efficacy of topical herbal derivates. Tvarijonaviciute et al. studied a population of 55 patients with OLP [75]. They treated 26 patients with 2% chamomile (*Chamaemelum nobile)* gel and 29 with placebo, applied three times a day for 4 weeks. For each patient, salivary total antioxidant status was evaluated before and after 4 weeks of treatment, by four methods: two TAC (6-hydroxy-2,5,7,8-tetramethylchroman-2-carboxylic acid) equivalent antioxidant capacity methods (TAC1 and TAC2), cupric reducing antioxidant capacity (CUPRAC), and ferric reducing ability of plasma (FRAP). Higher levels of FRAP were detected in the placebo group (*p* < 0.05). Moreover, significant correlations were observed between xerostomia and TAC1, TAC2, and CUPRAC, and between pain and drainage and TAC1, CUPRAC, and FRAP.

Another group of authors verified the utility of anthocyanins, extracted from grape skin, for topical treatment of OLP, comparing them with clobetasol propionate-neomycin-nystatin cream, used as control [76]. That study included 52 patients with OLP: 38 patients (17 cases and 21 controls) presented erosive OLP and 14 (9 cases and 5 controls) presented non-erosive OLP. In general, all patients were followed for 6 months, and the authors analyzed the oral mucosa involvement score, studied the affected areas morphometrically, and evaluated the therapeutic response time, the evolution of pain, and the relapse rate between the two groups. The findings indicate a favorable response to local treatment with anthocyanins in erosive OLP patients, better than controls, according to involvement score and morphological characteristics, while no statistically significant differences were found in therapeutic response time and the evolution of pain. In the non-erosive OLP group, the authors observed an improvement in pain relief, while the remaining analyzed variables showed no significant difference between treatments.

Finally, Veneri et al. investigated the effectiveness of ozonized water in erosive OLP [77] in patients who also received conventional corticosteroid therapy. They enrolled 51 patients and randomized them into two groups, one receiving the test agent and the second receiving placebo treatment for 4 weeks. For all patients in the first group, there was a higher significant improvement of sign and pain scores (improvement rates: Thongprasom score 92.2% vs. 28%, *p* < 0.05; VAS pain score 76.9% vs. 32%; *p* < 0.05). Candidiasis and relapse were also recorded, but the differences were not statistically significant between the two groups.

## 4. Discussion

The treatment of chronic inflammatory skin conditions is generally based on topical therapies, such as corticosteroids and calcineurin inhibitors, and/or systemic therapies, including antihistamines, corticosteroids, retinoids, and immune system suppressants [78,79,80]. Recently, a deeper understanding of the pathogenesis of these diseases has led to the development of novel therapeutic options targeting key molecules of the immune system or the pathological pathways [81]. For example, the last years have witnessed a substantial revolution in the treatment of many skin diseases (e.g., bullous diseases, urticaria, atopic dermatitis, hidradenitis suppurativa, and psoriasis) with biotechnological drugs [82]. The success of these new therapies lies in their great selectivity of action, which in most cases provides significant therapeutic efficacy in a short time with reduced side effects compared to traditional therapies [83,84,85,86].

### 4.1. Antioxidants Potential

In this phase of the therapeutic revolution, studies on drugs that target OS have become increasingly frequent [87]. Antioxidant therapies provide useful support in many diseases, e.g., hypertension and kidney disease [88,89], digestive and neurological pathologies [90,91], and in oncological patients [92]. Supplementation with antioxidant drugs seems to also be helpful in the dermatological field [32]. In particular, the main indications for antioxidants in dermatology include prevention and repair of UV photodamage and photoaging, the induced UV melanogenesis that occurs in melasma, and photo-carcinogenesis [93,94,95]. Moreover, the use of oral or topical antioxidants in the treatment of inflammatory dermatoses can neutralize excess ROS [32,33]. In this review, we focused on three important immune-related skin diseases: psoriasis, vitiligo, and lichen planus, but the importance of OS balance is also fundamental in other inflammatory skin conditions, such as acne. Acne, as a chronic inflammatory disorder of the pilosebaceous unit, may be a perfect example of how ROS are drivers of early inflammation. Impaired follicular walls of sebaceous glands contain nitrous oxide, superoxide, and hydroxyl. *P. acnes* participates in ROS production [96]. Oral and locally delivered antioxidants may reduce inflammation; studies have evaluated polyphenols, including alpha-mangotin, curcumin, ellagic acid, epigallocatechin 3-gallate, icariin, ganhuangenin, myricetin, resveratrol, myricitrin, schisandrin, and terchebulin with success [97,98]. However, data from controlled trials are limited [99,100]. Finally, single studies describe the safety and efficacy of topical antioxidants [101,102,103,104].

**Figure 1:**
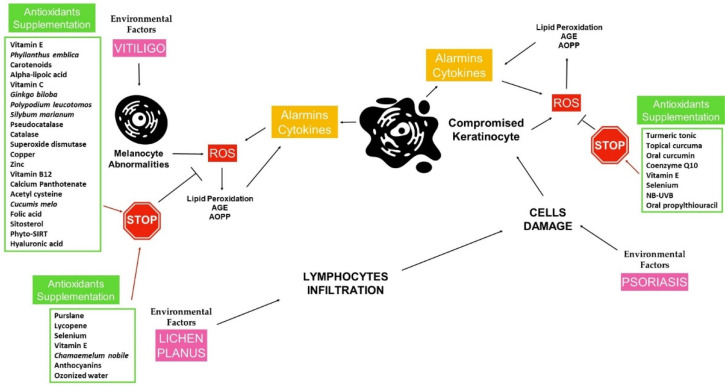
Environmental factors have a key role in triggering a cascade role, leading to the damage of skin cells. These events have a fundamental role in the oxidative stress/inflammation axis, which amplifies skin alteration, resulting in the worsening of the cutaneous disorders. Pro-inflammatory cytokines, together with alarmins and ROS, sustain the loop at the base of vitiligo, psoriasis, and lichen planus. In this scenario, the molecules reviewed seem to have a potential antioxidant effect.

As mentioned above, our review focused on the use of therapies with antioxidant potential in three chronic inflammatory skin diseases: PSO, vitiligo, and LP. Few literature studies were analyzed, based on the aforementioned inclusion criteria. Interesting observations can be drawn from the data, as shown in Figure 1.

### 4.2. Nutraceuticals as Antioxidants

Several clinical studies analyzed the efficacy of herbal derivates in inflammatory skin diseases. Curcumin was one of the main antioxidant drugs used. Six articles pointed out the high efficacy in moderate to severe PSO with both topical and oral supplementation [36,37,38,39,40,41]. Only one group of authors did not demonstrate a significant improvement in PSO patients with the use of oral *Curcuma* [42]. Curcumin (diferuloylmethane) is the active component of turmeric (*Curcuma longa*), a polyphenolic plant material. Recent studies have demonstrated that oral curcumin may be effective in patients with type 2 diabetes mellitus, metabolic syndrome, cardiac disease, and cancer. In fact, curcumin may downregulate inflammatory targets including lipoxygenase and cyclooxygenase-2, and inducible nitric oxide synthase. It may also inhibit many inflammatory cytokines, including TNF-α and interleukin-1, -2, -6, -8, and -12. Moreover, it has been hypothesized that curcumin may suppress NF-κB, on the whole playing an anti-inflammatory and antioxidant role [105,106].

Another polyphenol used in the selected studies is silymarin, a complex of the flavonolignans silibinin, isosilibin, silychristin, and silydianin obtained from the seed coats of milk thistle (*Silybum marianum*). It acts as a scavenger of free radicals and prevent peroxidation processes [107]. In fact, intake of silymarin extract was associated with a reduction in depigmented areas in vitiligo patients [58].

Anthocyanins are also polyphenols. Numerous reports have demonstrated their high bioavailability and protection against diseases induced by free radicals [108]. In particular, OLP patients showed a favorable response to local treatment with anthocyanins from grapes [76].

*Ginkgo biloba* extract contains unique polyphenol compounds such as terpenoids (ginkgolides and bilobalides), flavonoids, and flavanol glycosides. It has anti-inflammatory, immunomodulatory, and antioxidant properties. In particular, it attenuates oxidative stress in macrophages and endothelial cells [109]. It had been tested in several diseases, e.g., cardiovascular, neurological, endocrinological, and cutaneous disorders [110]. In a group of patients with vitiligo, treatment with *G. biloba* extract resulted in marked to complete repigmentation, arresting the progression of disease [56].

*Polypodium leucotomos* is a tropical fern plant, chemically composed of phenolic compounds such as p-coumaric, ferulic, caffeic, vanillic, and chlorogenic acids, which seem to have antitumoral and anti-inflammatory properties. It may stimulate IL-10 production, promoting a shift from a T-cell type 1 to a T-cell type 2 cytokine profile. Moreover, it seems to have antioxidant properties, reducing ROS and lipid peroxidation [111]. These functions may be involved in protecting against PUVA- and UV-induced damage to human skin, decreasing erythema, and preserving Langerhans cells [112]. A clinical trial showed that the combination of *P. leucotomos* with NB-UVB led to increased repigmentation, mainly in the head and neck area. In fact, this herbal derivate may induce reduction in TNF-α and IL-6, both of which are increased in vitiligo patients and known inhibitors of human melanocyte proliferation and melanogenesis [57,113].

*Portulaca oleracea L*., or purslane, is another herbaceous weed whose extracts can be used for their anti-inflammatory, antiulcerogenic, antifungal, and antioxidant properties. Indeed, it contains several biologically active compounds, such as omega-3 fatty acids, minerals, β-carotene, melatonin, and vitamins A, C, and E [114]. In about 83% of OLP patients in one study, treatment with oral purslane resulted in partial to complete clinical improvement [71]. Chamomile (*Chamaemelum nobile)* has been also studied as topical therapy for OLP. It contains numerous active flavonoids such as alpha bisabolol, azulene, matricin, and chamazulene, all of which exert antioxidant, anti-inflammatory, antispasmodic, antibacterial, and immunoregulatory activity [115]. Indeed, chamomile has been used for the treatment of oral diseases, including OLP. Application of chamomile gel resulted in pain relief and decreased burning sensation and itching, but this topical therapy was not associated with improvement of the disease [75].

In addition to plant extracts, other micronutrients have been analyzed, such as vitamins, trace elements, and carotenoids. Vitamin E is able to neutralize singlet oxygen in the cell membrane and prevent lipid peroxidation [116]. It seems that oral vitamin E supplementation could be useful in yellow nail syndrome, vibration disease, epidermolysis bullosa, cancer prevention, claudication, cutaneous ulcers, and collagen synthesis and wound healing [117]. Vitamin E was shown to be efficient in a group of PSO patients, in combination with two other antioxidant drugs, coenzyme Q and Se [43]. On the contrary, the combination of vitamin E with Se resulted in no disease improvement [44]. Coenzyme Q acts by reducing the production of free radicals and regenerating vitamin E. It also reduces keratinocyte DNA damage, UVA-induced metalloproteinase production in the fibroblasts, and mitochondrial oxidative damage [118]. Se is an essential trace element that participates in antioxidant defense and redox state regulation [119]. Some authors have shown that Se delays skin aging by protecting keratinocyte stem cells against UVA-induced cytotoxicity [120]. Supplementation with Se resulted in partial improvement in OLP patients; the improvement was similar with both topical and systemic Se and similar to that obtained with topical corticosteroids. Patients who were given Se had a sustained effect, and had lower pain and clinical lesion size scores compared with those who received topical corticosteroids. Taken together, these data suggest that this antioxidant therapy could be a valid alternative to classic therapy with corticosteroids. Moreover, antioxidants can be used as steroid-sparing treatment and are not associated with an increased risk of secondary candidiasis [73].

Oral intake of vitamin E was also effective in vitiligo patients. In particular, NB-UVB is the first-line treatment for vitiligo, but it has some side effects, such as erythema. The combination of phototherapy with vitamin E resulted in not only better repigmentation than NB-UVB alone, but also reduced UVB-induced erythema [53]. Moreover, the combination of vitamin E with other antioxidants, such as alpha-lipoic acid, vitamin C, *Phyllantus emblica*, and carotenoids, resulted in reduced vitiligo-associated OS [54,55]. Vitamin C removes free radicals and regenerates oxidized vitamin E, while lipoic acid can improve the repair mechanisms of the endogenous antioxidant system and neutralize free radicals [121]. Carotenoids seem to have both anti-inflammatory and antioxidant properties. They modulate the enzymatic activity of lipoxygenases to quench singlet oxygen, scavenge free radicals (including superoxide anions and hydroxyl radicals), and protect cell membranes and tissues against the effects of UV light [122]. Lycopene is among the group of carotenoids. The beneficial role of supplementation with oral lycopene in patients with leukoplakia has been demonstrated [123]. Moreover, a clinical trial on patients with OLP showed that oral supplementation with lycopene resulted in total or partial relief of symptoms in 50% or more of patients [72]. Taken together, these data suggest that oral lycopene could be an important tool in the management of certain premalignant oral lesions and conditions. Additionally, application of topical vitamin E was analyzed in patients with OLP, and proved to be effective in reducing the dimension of the lesions [74].

### 4.3. Other Antioxidants Therapies

Another class of drugs is topical antioxidants, such as PC-KUS or CAT and SOD cream. In patients with vitiligo, in order to compensate for low catalase levels, PC-KUS was introduced for removal of overproduced epidermal H_2_O_2_ [60]. Indeed, it was demonstrated that reduced epidermal H_2_O_2_ levels correlated with cessation of disease in 95% of patients in conjunction with extensive repigmentation [124]. PC-KUS requires UV light for full activation [125]. Two clinical trials demonstrated the efficacy of this cream plus NB-UVB [59,60], while another two did not confirm any significant improvement of lesions in vitiligo patients [61,62]. Four other studies analyzed the efficacy of topical CAT in association with SOD; two showed improvement of lesions [63,67], while two did not [64,65]. Another study analyzed the antioxidant properties of SOD cream in association with other antioxidants (Cu, Zn, vitamin B12, calcium pantothenate or acetyl cysteine, and phenylalanine) [66,68]. Furthermore, a clinical trial evaluated the antioxidant properties of a cream containing folic acid, phenylalanine, sitosterol, and hyaluronic acid [69]. In summary, what emerges from all these works is that the use of these antioxidant creams in vitiligo patients has variable results, from moderate to not significant, compared to conventional therapy or placebo. However, the combination with phototherapy (NB-UVB or excimer laser) or topical corticosteroids significantly improves repigmentation in vitiligo patients. In particular, it results in a shorter response time and a higher grade of repigmentation. Ultimately, it may reduce the possible side effects of UV irradiation (e.g., erythema, itching, mild burning, or pain) [126] or topical corticosteroids (e.g., dermal atrophy, acne, rosacea, telangiectasia, ecchymoses, or striae).

With regard to treatment with corticosteroids in OLP, it has been observed that the combination with ozonized water improved lesions in the oral cavity and associated symptoms [77]. Prolonged treatment with topical corticosteroids may be associated with side effects such as dysgeusia, tachyphylaxis, oral mucosa thinning, systemic absorption, and secondary candidiasis, and with increased risk of malignancies due to immune system suppression. It is therefore essential to evaluate safer alternative therapeutic strategies. Among non-pharmacological therapies, ozone use has been increasing due to its many favorable characteristics, including immune modulation, pain relief, antimicrobial effect, wound healing, and, not least, antioxidant properties.

Some articles analyzed the antioxidant properties of conventional therapies used in the treatment of inflammatory skin diseases. One article pointed out that MTX had no effect on PSO-associated OS [47]. MTX has immunosuppressive effects with mechanisms of action related to ROS generation. In particular, it quenches CAT and SOD levels and TAC. However, despite the evidence of MTX-induced OS, other clinical evidence suggests that MTX may have antioxidant activity. Some authors have shown that managing rheumatic disease with MTX reduces inflammation and OS levels [127,128].

Relating to treatment with biological drugs, two studies demonstrated a significant improvement of OS in PSO patients, particularly with two TNFα-I drugs, infliximab and etanercept [51,52]. The two therapies block the inflammatory response by directly binding TNFα or interfering with the binding of TNFα to its receptors, respectively. It was observed that injection of these drugs in PSO patients was associated with reduced levels of markers of lipid peroxidation, decreased susceptibility to copper-induced lipid peroxidation, and increased TAC [51]. These results were also confirmed by other studies, which noted that TNFα-I may reduce OS in other inflammatory diseases, such as ankylosing spondylitis and rheumatoid arthritis [129]. Indeed, TNFα can induce ROS production from neutrophils through the phagocytic nicotine adenine dinucleotide phosphate (NADPH) oxidase pathway. Moreover, it can reduce antioxidant levels, including GSH and NADPH. Finally, TNFα, in association with other cytokines, can enhance O_2_ generation [51,130].

One group of authors analyzed the antioxidant effects of tacrolimus and pimecrolimus [70], two topical calcineurin inhibitors recommended as first-line treatment for limited forms of vitiligo [131]. These two drugs downregulate proinflammatory cytokines such as TNFα and induce anti-inflammatory cytokines such as IL-10. Moreover, they induce melanocyte migration and proliferation and melanogenesis. Tacrolimus has shown protective effects in experimental in vitro models of oxidative stress by increasing GSH levels, inhibiting arachidonic acid release, and increasing cellular TAC [70].

Finally, in some studies evaluated the antioxidant power of an antithyroid drug. Three studies analyzed the effect of supplementation with PTU in PSO patients [48,49,50]. PTU has immunomodulatory effects and antioxidant potential in addition to its inhibitory effect on thyroid hormone synthesis [48]. In previous studies, a possible immunosuppressive effect of PTU on lymphocytes from normal volunteers and patients with Graves’ disease was demonstrated [132]. In PSO patients, PTU produces significant clinical improvement, with reduced epidermal thickness, and leads to decreased PASI scores.

Regarding physical therapy, two groups of authors analyzed the role of NB-UVB in OS in PSO patients [45,46]. UVB seemed to have not only a pro-oxidant, but also an antioxidant effect. In particular, the authors noted that, under NB-UVB, psoriatic plaques showed significant improvement (decreased PASI score), and phototherapy induced decreased ROS and MDA levels. Reduced Se and Zn levels were also observed, but this did not negatively affect the final improvement of lesions.

### 4.4. Pharmacological Safety

An important aspect to consider in all treatments with antioxidant potential is the pharmacological safety [32]. As pointed out by many studies, using oral or topical antioxidants does not replace consuming a diet with adequate amounts of fruits and vegetables. When antioxidant supplements are used, it is fundamental that, during treatment, the concentrations of exogenous antioxidants in plasma become close to the physiological ones, in order to reduce the risk of toxicity or even interactions with drugs the patient takes [35]. In particular, in the majority of cases, antioxidants can induce adverse effects when used indiscriminately. For example, there is a high risk of hypervitaminosis in patients with renal dysfunction, due to reduced renal excretory function [133]. Interestingly, in our review, 8 trials out of 42 did not report adverse effects (Table 1, Table 2 and Table 3). The same antioxidant treatments were associated in different trials with mild or no adverse effects. In particular, curcuma did not have collateral effects only when topically applied. On the contrary, mild adverse reactions were described with oral intake of curcuma (gastrointestinal upset or transient erythema). Oral intake of vitamin E and herbal derivatives was also associated with similar symptoms. As reported in the literature, indiscriminate use of tocopherol can inhibit glutathione-S-transferase, responsible for removing cytotoxic compounds related to tumorigenesis in the skin [134]. Cutaneous adverse reactions, such as contact dermatitis, eczematous rash, acneiform eruption, or erythema, were more frequently described after topical application of antioxidant cream. However, in 17 trials (40% of the total), data on adverse effects are missing, precluding an accurate comparison among the various trials. Comparison is also made difficult by the small number of studies on individual drugs. Moreover, the number of patients included in the trials was not consistently high in the majority of cases (fewer than 50 patients, except for eight trials). Furthermore, in some studies the placebo or control group was missing (*n* = 7, 17% of the total).

## 5. Conclusions

While the use of antioxidants in the treatment of inflammatory skin diseases may appear to be of interest, an analysis of the literature suggests that further studies should be carried out on this category of molecules. It will be essential to consider larger populations in these studies, also using internationally validated scales, to be able to compare results and clinical efficacy.

Notably, in most cases, antioxidants alone are not able to induce significant clinical changes in the aspect and/or course of the disease, except perhaps in mild forms; they must be used in conjunction with standard pharmacological treatments to achieve measurable results. Another important element that deserves to be better explored is that combinations of antioxidant treatments seem to be more effective by exploiting the synergistic effect of the various molecules, which act differently on oxidative stress.

## Figures and Tables

**Table 1:** Summary of studies on the efficacy of treatments with antioxidant potential in psoriasis.

Treatment	Authors-Year	N° of pt	Study Design	Grade of PSO	Formulation and Dose	Duration of Exposure–Treatment	Degree of Improvement	Adverse Effects
**Curcuma**	Heng et al., 2000	10	Four-armed randomized controlled clinical trial	Moderate	Topical, once daily	8 w	High	Data missing
	Bahraini et al., 2018	40	Randomized, double-blind, placebo-controlled, prospective clinical trial	Mild to moderate	Topical, twice daily	9 w	High	None
	Asin-Llorca et al., 2006	20	Pilot non controlled clinical trial	Moderate to severe	Oral 600 mg/d + UVA	8 w	Total	Data missing
	Kurd et al., 2008	12	Phase II, open-label, Simon’s two-stage trial	Moderate to severe	Oral 4.5 g/d	16 w	Low	Mild (gastrointestinal upset, heat intolerance/hot flashes, respiratory events, and musculoskeletal and neurological events)
	Antiga et al., 2015	49	Phase III, single-dose, randomized, double-blind, placebo-controlled clinical trial	Moderate to severe	Oral 2 g/d+ topical steroid	12 w	High	Diarrhea; papular eruption on the face and nausea
	Carrion-Gutierrez et al., 2015	21	Phase IV randomized, double-blind, placebo-controlled, pilot clinical trial	Moderate to severe	Oral 600 mg/d+ visible blue light	10 w	High	Mild (not specified in detail)
	Ramirez-Bosca et al., 2016	24	Phase IV, randomized, with third party blind evaluation, uncenter, open pilot	Moderate to severe	Oral 600 mg/d+ visible blue light	12 w	High	Rare and mild (not specified in detail)
**Coenzyme Q10,** **Vitamin E,** **Selenium**	Kharaeva et al., 2009	30 EP 28 PSA	Randomized double-blind placebo-controlled clinical study	Severe	Ubiquinone acetate 50 mg/d Alpha tocopherol 50 mg/d Selenium aspartate 48 µg/d	1 m	Faster in PSA Slow in EP	Data missing
**Vitamin E,** **selenium**	Fairris et al., 1989	69	Randomized, double blind, placebo-controlled trial	Moderate to severe	Oral Se 600 µg/d Oral vitamin E 600 UI/d	12 w	None	None
**NB-UVB**	Wacewicz et al., 2017	60	Case control study	Moderate	20 sessions	-	High	Data missing
	Darlenski et al., 2018	22	Case control study	Mild to moderate	14 sessions	-	MIld	Data missing
**MTX**	Akbulak et al., 2017	21	Case control study	Moderate to severe	10–15 mg/w	12 w	None	Data missing
**PTU**	Elias et al., 1993	10	Open, single-centre study	Moderate to severe	Oral 300 mg/d	8 w	High	Mild metallic taste, higher TSH levels
	Elias et al., 1994	9	Double blind, placebo-controlled trial	Moderate to severe	Topical 60 g in 5% lotion twice/w	4–8 w	High	None
	Köse et al., 2002	30	Randomized, controlled clinical trial	Moderate	Oral 100 mg/d	8 w	High	Higher TSH levels within the reference range
**Etanercept**	Bacchetti et al., 2013	23	Open label, controlled and non-randomized clinical trial	Moderate to severe	I phase 50 mg biweekly, II phase 25 mg biweekly	I phase 12 w II phase 12 w	High	Data missing
**Infliximab**	Barygina et al., 2013	29	Randomized, double blind, controlled trial	Moderate	5 mg/Kg every 8 w	6 m	Significant improvement	Data missing

*Abbreviations*: d = day(s), m = month(s), w = week(s), pt = patients, EP = erythrodermic psoriasis, MTX = methotrexate, NB-UVB = narrow band UVB, PSA = psoriatic arthritis, PTU = propylthiouracil, and Se = selenium.

**Table 2:** Summary of studies on the efficacy of treatments with antioxidant potential in vitiligo.

Treatment	Authors-Year	N° of pt	Study Design	Type of Vitiligo	Formulation and Dose	Duration of Exposure–Treatment	Degree of Improvement	Adverse Effects
Vitamin E	Elgoweini et al., 2009	24	Comparative, prospective clinical trial	Vulgaris	Oral alpha-tocopherol 400 IU, once daily	6 m	Excellent improvement in 50% of pt	Mild erythema
Vitamin E *plus Phyllanthus emblica* and carotenoids	Colucci et al., 2014	130	Comparative, prospective clinical trial	Vulgaris	Tab containing *P. emblica* (100 mg), vitamin E (4.7 mg), and carotenoids (10 mg); 1 tab 3 times/d	6 m	Mild improvement	None
Vitamin E *plus* alpha-lipoic acid and vitamin C	Dell’Anna et al., 2007	35	Prospective, randomized, double-blind, placebo-controlled multicentre study	Vulgaris	Tab containing 20 mg vitamin E, 50 mg alpha-lipoic acid, 50 mg vitamin C; 2 tab/d	8 m	Excellent improvement in 47% of pt	Minimal (not specified in detail)
*Ginkgo biloba*	Parsad et al., 2003	52	Double-blind, placebo-controlled trial	Vulgaris, focal and acrofacial	Tab containing *G. biloba* extract 40 mg; 1 tab 3 times/d	6 m	Excellent improvement in 40% of pt	Mild nausea
*Polypodium leucotomos*	Middelkamp-Hup et al., 2007	50	Prospective double-blind randomized placebo-controlled clinical trial	Vulgaris	Tab containing 250 mg of *P. leucotomos*; 1 tab 3 times/d	26 w	Significant improvement in head and neck area	Mild gastrointestinal complaints
*Silybum marianum*	Jowkar et al., 2019	34	Prospective, double-blind randomized controlled clinical trial	Not specified	Tab containing 140 mg of silymarin; 1 tab twice daily	3 m	Significant improvement	Data missing
Pseudocatalase	Schallreuter et al., 1995	33	Open and uncontrolled clinical trial	Vulgaris, acrofacial, focal and segmental	Cream twice daily	15.3 m	Excellent in 90% of pt with acrofacial or focal vitiligo. Partial improvement in pt with segmental and vulgaris vitiligo. No improvement in pt with lesion on fingers and feet.	Contact dermatitis to para-aminobenzoic acid ester used as preservative
	Schallreuter et al., 2002	59	Randomized three arm study	Vulgaris and acrofacial	Cream twice daily	21 d	Significant improvement	Data missing
	Patel et al., 2002	26	Open, single-centre study	Acrofacial	Mousse twice daily	6 m	Variable improvement	Eczematous rash, acneiform eruption
	Bakis-Petsoglou et al., 2009	32	Double-blind, placebo-controlled, randomized, single-centre trial	Acrofacial	Cream twice daily	6 m	No statistically significant improvement	Sweating, pruritus, erythema, and inflammation
Catalase *plus* superoxide dismutase	Schallreuter et al., 2005	6	Open and uncontrolled clinical trial	Facial	Gel twice daily	4 m	No improvement	Data missing
	Kostovic et al., 2007	22	Open and uncontrolled clinical trial	Vulgaris, acrofacial, segmental and focal	Gel twice daily	6 m	Good improvement in 57.9% of pt, especially on the face and neck	Mild erythema at the application site
	Sanclemente et al., 2008	25	Randomized, matched-paired, double-blind trial	Vulgaris	Cream twice daily	10 m	Improvement in 56.5% of pt	Mild local erythematous papular rash
	Yuksel et al., 2009	30	Randomized clinical trial	Not specified	Gel twice daily	6 m	None	None
Superoxide Dismutase *plus* Copper, Zinc, Vitamin B12 and Calcium Panthotenate	Soliman et al., 2016	30	Comparative, prospective, randomized study	Vulgaris	Hydrogel once daily	24 sessions (3 times/w)	Significant improvement	None
Acetyl cysteine, phenylalanine and *Cucumis melo* extract	Buggiani et al., 2012	149	Four-armed open study	Symmetrical vitiligo	Gel twice daily	12 w	Marked to excellent improvement in about 70% of pt	None
Folic acid, phenylalanine, sitosterol, Phyto-SIRT and hyaluronic acid	Leone et al., 2015	10	Pilot randomized, investigator-blinded, and half-side comparison trial	Vulgaris	Cream twice daily	3 m	Significant improvement	Mild erythema
Tacrolimus 0,1% or pimecrolimus 1%	Lubaki et al., 2009	20 + 20	Randomized double-blind placebo control study	Acrofacial and vulgaris	Ointment or cream twice daily	7 m	Tacrolimus treatment: moderate to good improvement in 35% of pt, mild in 45% of pt, none in 20% of pt. Pimecrolimus treatment: good improvement in 70% of pt with acrofacial vitiligo, mild in vulgar vitiligo	Transient pruritus

*Abbreviations*: d = day(s), m = month(s), w = week(s), pt = patients, and tab = tablet(s).

**Table 3:** Summary of studies on the efficacy of treatments with antioxidant potential in lichen planus.

Treatment	Authors-Year	N° of pt	Study Design	Type of OPL	Formulation and Dose	Duration of Exposure–Treatment	Degree of Improvement	Adverse Effects
Purslane	Agha-Hosseini et al., 2010	37	Randomized double-blind placebo-controlled trial	Not specified	Cap with 235 mg of Purslane extract; 1 cap/d	3 m	Partial	None
Lycopene	Saawarn et al., 2011	30	Prospective, randomized, double-blind, placebo-controlled study	Not specified	Cap with 8 mg of Lycopene; 1 cap/d	8 w	High	Data missing
Selenium	Qataya et al., 2020	32	Three-armed randomized controlled clinical trial	Erosive	Tab containing 200 μg of Se: 2 tab/d; gel containing 1.4 mg of Se: twice daily	6 w	Partial	Data missing
Vitamin E	Bacci et al., 2016	34	Randomized, double-blind, crossover study	Reticular	Gel 3 times/d	10 w	High	Data missing
*Chamaemelum nobile*	Tvarijonaviciute et al., 2018	55	Randomized, double-blind, parallel group study	Not specified	Gel 2%, 3 times/d	4 w	Partial	Data missing
Anthocyanins	Rivarola de Gutierrez et al., 2014	27	Prospective, non-randomized, controlled study	Erosive and non-erosive	100 mg doses diluted in 5 mL of water; three times/d	6 m	Partial	Data missing
Ozonized water	Veneri et al., 2020	51	Randomized placebo-controlled study	Erosive	double-distilled water plus ozone (ratio being 2:3); 4 times/d, twice weekly	4 w	High	Data missing

*Abbreviations*: d = day(s), m = month(s), w = week(s), cap = capsule(s), pt = patients, tab = tablet(s), and Se = selenium.

## Abbreviations

Advanced glycation end products (AGEs); advanced oxidation protein products (AOPPs); catalase (CAT); copper (Cu); cupric reducing antioxidant capacity (CUPRAC); erythrodermic psoriasis (EP); ferric reducing ability of plasma (FRAP); glutathione (GSH); glutathione peroxidase (GPX); glutathione-S-transferase (GST); interleukin (IL); lichen planus (LP); malondialdehyde (MDA); methotrexate (MTX); narrow band ultraviolet B (NB-UVB); oxidative stress (OS); pseudocatalase cream (PC-KUS); paraoxonase (PON); psoriasis (PSO); propylthiouracil (PTU); psoralen plus UVA therapy (PUVA); reactive oxygen species (ROS); total antioxidant capacity (TAC); tumor necrosis factor α inhibitors (TNFα-I); and Visual Analogue Scale (VAS).
